# Oestrogen-induced epithelial-mesenchymal transition (EMT) in endometriosis: Aetiology of vaginal agenesis in Mayer-Rokitansky-Küster-Hauser (MRKH) syndrome

**DOI:** 10.3389/fphys.2022.937988

**Published:** 2022-12-13

**Authors:** Too Lih Yuan, Nadiah Sulaiman, Abdul Ghani Nur Azurah, Manira Maarof, Rabiatul Adawiyah Razali, Muhammad Dain Yazid

**Affiliations:** ^1^ Centre for Tissue Engineering and Regenerative Medicine, Faculty of Medicine, Universiti Kebangsaan Malaysia Medical Centre, Cheras, Malaysia; ^2^ Department of Obstetrics and Gynaecology, Faculty of Medicine, Universiti Kebangsaan Malaysia Medical Centre, Cheras, Malaysia

**Keywords:** endometriosis, epithelial-mesenchymal transition, Mayer-Rokitansky-Küster-Hauser syndrome, oestrogen, Wnt4

## Abstract

Endometriosis occurs when endometrial-like tissue forms and grows outside the uterus due to oestrogen-induced epithelial-mesenchymal transition in the female reproductive tract. Factors that suppress this event could become potential therapeutic agents against disease occurrence and progression. However, an overview of these studies is still lacking. This review assessed the impact of a number factors on oestrogen-mediated epithelial-mesenchymal transition in the emergence of several diseases in the female reproductive tract, primarily endometriosis. The association between epithelial-mesenchymal transition and Mayer-Rokitansky-Küster-Hauser (MRKH) syndrome was also investigated. Oestrogen, Wnt4 and epithelial-mesenchymal transition were chosen as keywords in Scopus, PubMed, and Web of Science searches performed on 28th June 2021. Study selection was refined to cancer-irrelevant, English, original articles published between years 2011–2021. The full-text assessment was carried out for topic-related articles after title and abstract screening. Included studies were summarised and assessed for their risk of bias using the Office of Health Assessment and Translation tool. In this review, 10 articles investigating oestrogen and epithelial-mesenchymal transition in the female reproductive tract were summarised and classified into two groups: seven studies under ‘factor’-modulated epithelial-mesenchymal transition and three studies under ‘factor’-manipulated oestrogen-induced epithelial-mesenchymal transition. The current evidence proposes that epithelial-mesenchymal transition is one of the prime causes of reproductive-related disease. This event could be mediated by distinct stimuli, specifically oestrogen and Wnt4 aberration. The results of this review suggest that oestrogen and Wnt4 participate in epithelial-mesenchymal transition in vaginal epithelial cells in MRKH syndrome, adopting from the theories of endometriosis development, which could therefore serve as a foundation for novel target treatment, specifically related to vaginal epithelialisation, to ensure better surgical outcomes.

## 1 Introduction

Endometriosis (EM) is a benign gynaecological disease affecting approximately 6–10% of reproductive age women worldwide (Li et al., 2021). It is a condition where the tissue that usually lines the uterus grows outside of the uterine cavity and appears as endometrial glands and stroma-like lesions (Pontikaki et al., 2016; Yang and Yang, 2017). EM is also defined as an oestrogen-dependent inflammatory disorder, and studies have been continuously executed to develop suitable treatments for EM in response to oestrogen (E2) (Mai et al., 2019). In a study conducted by Pellegrini and others, the mRNA expression of oestrogen receptors (ERα and Erβ) in human ectopic endometrial tissue was found to be significantly upregulated compared to normal and eutopic endometrial tissues (Pellegrini et al., 2012). Similarly, Matsuzaki and others demonstrated that both eutopic endometrium and endometriotic tissues obtained from patients displayed high mRNA levels of ERα and Erβ (Matsuzaki et al., 2001). These findings suggest that E2 is likely to exert a promoting effect during EM development by binding to its receptors (Trukhacheva et al., 2009).

Epithelial-mesenchymal transition (EMT) is a reversible transition of cell features from an epithelial to mesenchymal phenotype, which provokes cellular changes in morphology, inflammation (Sisto et al., 2019), invasion, migration (Furuya et al., 2017) and proliferation (Meng et al., 2016). Epithelial cells undergoing the transition process often transform from a cobblestone shape to a spindle shape (Chen et al., 2017). Importantly, the transition may not be permanent, as transformed mesenchymal cells could convert back to epithelial derivatives *via* mesenchymal-epithelial transition (MET) (Kalluri and Weinberg, 2009). Generally, EMT is known to be crucial for embryogenesis, wound healing and cancer, and thus has been categorised into three subtypes accordingly (Kalluri and Weinberg, 2009). Many studies have correlated EMT with the pathogenesis and development of EM (Matsuzaki and Darcha, 2012; Xiong et al., 2016; Lin et al., 2019; Zhang et al., 2019; Wang et al., 2020), inferring that the establishment of an endometriotic lesion would present the features of EMT, such as loss of cell polarity, disintegration of cell-cell junctions, increased in cell mobility, gain of N-cadherin expression and concomitant loss of E-cadherin expression (Bartley et al., 2014). As such, Proestling and others, who conducted a study using ectopic endometrial lesions from patients with EM, found that cadherin-1 mRNA expression was clearly downregulated while Twist-related protein 1 (TWIST1) mRNA expression was significantly increased (Proestling et al., 2015). Chen et al. revealed a significant decrease in E-cadherin expression and a significant increase in C-X-C motif chemokine 12 in the endometrial tissue of EM patients (Chen et al., 2020). Another finding presented by Yu et al. (2021) showed strong staining of N-cadherin and E-cadherin in endometriotic lesions and control endometria, respectively. These observations show that EMT participates in the development of EM, and is a workable indicator in tracing the aetiology of the disease.

To date, E2 has been extensively studied for its inductive roles in the occurrence of EMT in multiple diseases, particularly within the cancer field (Ding et al., 2006; Huang et al., 2007; Park et al., 2008; Mishra et al., 2015; Yoriki et al., 2019). Some studies have also suggested that external stimuli such as intracellular molecules and signalling pathways are partly involved in E2-induced EMT in cancer. As an example, Das and others overexpressed nuclear respiratory factor 1, a transcription factor in E2-treated MCF10 A cells, which resulted in the generation of highly invasive mesenchymal breast cancer stem cells *via* EMT (Das et al., 2018). However, studies investigating the influence of these stimuli on E2-induced EMT in the non-cancer field are still lacking. Hence, this review assessed how the EMT process can be modulated by these stimuli, including E2, mostly in EM development. This review also shows that E2 has possible interactions with Wnt4 in inducing EMT in vaginal epithelial cells, adopting the theories of EM pathogenesis. This further obstructs the process of vaginal epithelialisation in Mayer-Rokitansky-Küster-Hauser (MRKH) syndrome patients. The outcomes of this review are urgently needed as they might have clinical implications not only in terms of establishing new therapeutic agents against respective diseases, but also to foreseeably clarify the genesis of a number of conditions.

## 2 Materials and methods

### 2.1 Search methods

On 28 June 2021, a comprehensive search was conducted electronically in three databases, specifically PubMed, Scopus and Web of Science, to obtain relevant studies. The databases were searched for articles with the central keywords ‘oestrogen’, ‘epithelial-mesenchymal transition’ and ‘Wnt4’. Wnt family member 4 (Wnt4) was included to relate the EMT in MRKH as it was previously found to be mutated in an animal model and in MRKH patients. These keywords were decided on after a thorough discussion between the review authors, and several attempts were made with the modification of keywords and Boolean operators. The finalised and utilised terms were ‘Wnt4’ OR ‘oestrogen’ AND ‘epithelial to mesenchymal transition’ OR ‘EMT’.

### 2.2 Eligibility criteria

In addition to the application of the selected terms, the search was further restricted to a few criteria. The search was refined to 10-year limit for studies published from 2011 to 2021. Only English original articles were considered during the search. In contrast, articles respective to cancer research were unselected by adding the term ‘NOT cancer’ during the search. After the search was performed accordingly, the results were extracted to carefully screen the titles and abstracts for topic-related articles. As this review aimed for EMT regulation by E2 and/or distinct stimuli, articles unassociated to this aim were eliminated.

### 2.3 Selection of reviews

Three review authors worked separately to screen each retrieved study. Initially, the articles with titles irrelevant to the topic were removed, likewise when reviewing abstracts. Duplicates were all eliminated after the title assessment. The reviewers then explored the full texts of the selected articles to ensure data applicability for inclusion and to remove unrelated studies. Disagreement during the selection process was resolved through discussion among the review team.

### 2.4 Data extraction and synthesis

Three independently working reviewers extracted pertinent data from every chosen study. The request for full-text papers was conducted *via* ResearchGate for articles that were not publicly accessible. Data obtained from the chosen studies were summarised and thematically tabulated based on author, aim, disease/event, type of cell/tissue, treatment, findings and conclusions.

### 2.5 Risk bias assessment

Applying an adapted version of risk of bias tool which was the Office of Health Assessment and Translation (OHAT), three review authors independently assessed the risk of bias of the included studies (Ruszymah et al., 2020). This analysis tool entails the risk of bias in several domains: 1) selection bias; 2) performance bias; 3) detection bias; 4) attrition bias; 5) reporting bias. Studies were deemed as exhibiting a low risk of bias (+), high risk of bias (−), unclear risk of bias (?) or not applicable (NA) (Supplementary Table S1). Consensus was achieved *via* discussion between the review authors for any variance in the risk of bias analysis.

## 3 Results

### 3.1 Search results

**FIGURE 1:**
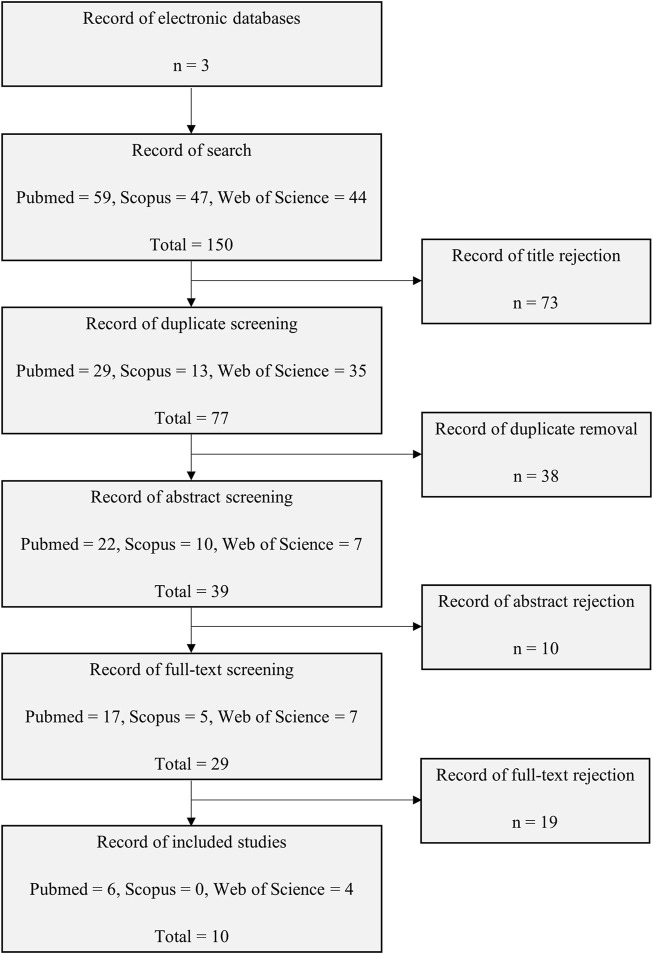
Flow chart of the study selection process from electronic databases of PubMed, Scopus, and Web of Science.

A total of 150 articles were yielded from the search of three databases mainly: 59 articles from PubMed, 47 articles from Scopus and 44 articles from Web of Science. Three review authors worked separately to screen each study retrieved to minimise bias while enhancing the quality of the search results. A total of 73 articles were eliminated based on title screening and 38 articles were found to be duplicated and thus removed. A further evaluation of the abstracts showed that 10 articles were irrelevant to either E2, Wnt4 or EMT. The remaining 29 articles were obtained and underwent a full-text assessment. The assessment resulted in 10 articles being selected for this review; the unselected studies were not focused on EMT. Figure 1 presents the flow chart of the selection process.

### 3.2 Study characteristics

All included studies in this review were published from 2011 to 2021 and were performed as *in vitro*-based research. Intriguingly, these studies are corresponded to female reproductive system which could be implying that Wnt4, E2 and EMT have imperceptible functions in female reproductive system. The data obtained from these studies were summarised and classified into two groups: seven studies under ‘factor’-modulating EMT and three studies under ‘factor’-manipulating E2-induced EMT. Collectively, the main disease investigated was EM, while three studies investigated intrauterine adhesion (IUA), polycystic ovary syndrome (PCOS) with/without endometrial hyperplasia and adenomyosis. Furthermore, the human cell type that was generally used in analysing the mechanisms of these diseases was endometrial cells. Only one study employed endometrial tissue. An overview of the selected studies is displayed in Table 1 and Table 2.

**TABLE 1:** Summary and classification of the articles on EMT modulation.

Type of Induction	Aim	Disease/ Event	Type of Cell/ Tissue	Treatment	Findings	Conclusion	References
Factor promoting EMT	1. To verify the role and expression of Rho family on proliferation and EMT in EM	EM	Human eutopic endometrial epithelial cells	Cells were co-cultured with RhoA and siRhoA lentiviral supernatants	1. Western blot analysis showed that RhoA enhancement attenuated E-cadherin expression and increased vimentin and N-cadherin expressions	RhoA/ROCK pathway was upregulated by E2/ERα/ERK pathway to promote EMT and proliferation, resulting in EM development	Huang et al. (2020)
	2. To assess the correlation and molecular mechanism between Rho family and E2 pathway				2. Transwell, EdU labelling, and CCK-8 assays revealed that RhoA promoted migration, cell division and proliferation		
					3. RhoA enhancement strengthened the effect of E2-promoting EMT in Western blot analysis		
	To investigate the function of E2 in cellular transition, migratory, and inflammatory responses of endocervical epithelial cells (EECs) and cervical stromal cells (CSCs)	Uterine contractions	1. Human endocervical epithelial cells	Cells were treated with 5,000 pg/mL E2 and 100 ng/ml LPS (an infection stimulus)	EECs and CSCs which were under untreated and LPS-treated conditions, E2 unaltered:	E2 probably supported EMT and localized inflammation in the presence of infection stimulus in migrating and remodelling cervical cells	Tantengco et al. (2021)
			2. Human cervical stromal cells		1. Immunocytochemical staining of CK-18 and vimentin expressions		
					2. Cell shape index based on cell shape analysis		
					3. Levels of EMT markers exhibited in Western blot analysis		
	1. To assess the effects of ZEB1 knockdown on growth, mobility, and invasion	EM	Ishikawa cells	Cells were transfected with recombinant shZEB1 lentivirus and ZEB1 promoter reporter gene recombinant vectors	1. ZEB1 downregulation suppressed cell proliferation, migration and invasion as shown in transwell and MTS analyses	E2 induced ZEB1 expression and contributed to EMT phenomenon in EM.	Wu et al. (2018a)
	2. To identify the effects of E2 on ZEB1 expression and promoter activity				2. Knockdown of ZEB1 induced E-cadherin while inhibited vimentin protein expressions as per Western blot results		
Factor inhibiting EMT	1. To study the effects of E2 on EMT in normal and fibrotic endometrium	IUA	1. Normal human endometrial epithelial cells.	1. Cells were cultured with 10, 20, 30 and 50 nM E2 for 24, 48 and 72 h	1. Induction of TGF-β1 in IUA cell model decreased epithelial markers and increased mesenchymal markers as confirmed by Western blotting. The effects were reversed in TGF-β1-induced fibrosis cells pre-treated with 30 nM E2	E2 inhibited TGF-β1-induced EMT by activating Wnt/β-catenin signalling pathway to prevent IUA occurrence and development	Cao et al. (2020)
	2. To investigate the function of E2 on TGF-β1-induced EMT and Wnt/β-catenin signalling in an IUA cell model		2. IUA cell model	2. Cells were exposed to 10, 30 and 50 ng/ml TGF-β1 for 24 h	2. RT-PCR analysis showed that mRNA expression levels of Wnt/β-catenin signalling molecules were increased in TGF-β1-induced fibrosis cells when E2 was added		
	1. To examine the effects of melatonin on migration, invasion and EMT in normal and endometriotic epithelial cells	EM	1. Normal endometrial epithelial cells (NECs)	1. Cells were treated with 1 mM melatonin	1. CCK-8 assay showed that melatonin decreased growth and E2-induced cell growth in EuECs and NEC.	By upregulating Numb expression and decreasing activity of Notch signalling pathway, melatonin might impede E2-induced migration, invasion and EMT in NECs and EuECs	Qi et al. (2018)
	2. To study possible signalling pathway involved		2. Endometriotic eutopic epithelial cells (EuECs)	2. Cell were cultured with/without 10 nM E2 for 24, 48 and 72 h	2. Migration and invasion assays revealed that melatonin significantly decreased migration and invasion in both cells		
					3. In Western blot, melatonin eliminated E2-induced vimentin and E2-downregulated Numb expressions in NEC.		
Factor altering EMT	1. To examine the expression and localization of MAPK signalling and EMT components in endometrium of non-PCOS and PCOS patients with and without EH	PCOS with and without EH	Endometrial tissue	Tissues were treated with 10 nM E2 and/or 20 mM metformin for 24 h	Immunoblot results showed treatment of metformin alone or in combination with E2 altered protein expressions of CK 8, Claudin 1, ZO-1, N-cadherin, Slug, Snail and α-SMA in endometrial tissues of PCOS patients	Abnormal regulation of EMT and MAPK signalling components impaired function and homeostasis of endometrial cells. Metformin regulated endometrial EMT protein expression in an E2-dependent manner.	Hu M et al. (2020)
	2. To identify the effects of metformin on EMT *in vitro*						
	1. To investigate the roles of miR200s and MALAT1 in ectopic endometrium	EM	1. Human endometrial epithelial cells	Cell were transfected with siRNA-MALAT1 and mir200c mimics	1. MiR200c reversed E2-mediated EMT.	E2-mediated MALAT1/miR200s expression to promote EMT in EM, which MALAT1 and miR200s exhibited significant reciprocal inhibition	Du et al. (2019)
	2. To examine the effects of E2 on EMT and MALAT1/ miR200s in both endometrial epithelial cells and Ishikawa cells		2. Ishikawa cells		2. MALAT1 knockdown attenuated E2-induced EMT.		
					3. E2 decreased E-cadherin expression and increased vimentin expression in a dose-dependent manner		

E2, oestrogen; EMT, epithelial-mesenchymal transition; EM, endometriosis; RhoA, transforming protein RhoA; EdU labelling, 5-ethynyl-2′-deoxyuridine labelling; CCK-8 assay, cell counting kit-8 assay; ERα, oestrogen receptor α; LPS, lipopolysaccharide; CK-18, cytokeratin-18; ZEB1, zinc finger E-box-binding homeobox 1; TGF-β, transforming growth factor beta 1; IUA, intrauterine adhesion; RT-PCR, real-time polymerase chain reaction; MAPK, mitogen-activated protein kinase; PCOS, polycystic ovary syndrome; EH, endometrial hyperplasia; CK 8, cytokeratin 8; ZO-1, zonula occludens-1; Slug, zinc finger protein SNAI2; Snail, zinc finger protein SNAI1; α-SMA, alpha-smooth muscle actin; MALAT1, metastasis associated lung adenocarcinoma transcript 1.

**TABLE 2:** Summary and classification of the articles on manipulation of E2-induced EMT.

	Aim	Disease/ Event	Type of Cell/ Tissue	Treatment	Findings	Conclusion	References
1	To assess the expression pattern and function of NRP1 in E2-induced EMT in endometrial cells of adenomyosis	Adenomyosis	Human endometrial cells (HEC-1-A)	1. Cells were infected with NRP1 and shRNA NRP1 retroviruses for 72 h	1. From RT-PCR results, NRP1 retrovirus infection decreased epithelial markers of CDH1 and occludin mRNA expression while increased mesenchymal markers of ACTA2 and N-cadherin mRNA expression in endometrial cells. The results were similar in Western blot analysis whereby expression of E-cadherin was reduced and α-SMA was enhanced	NRP1 participated in E2-induced EMT in promoting adenomyosis development	Hu R et al. (2020)
				2. Cells were treated with 0.1, 1 and 10 μM E2 for 24 h	2. Detected in both RT-PCR and Western blot, E2 treatment in endometrial cells promoted NRP1 protein and mRNA expressions in a dose-dependent manner		
					3. NRP1 shRNA obviously restored expressions of epithelial markers while lowered expressions of mesenchymal markers in E2-induced EMT in endometrial cells as presented in the results of RT-PCR and Western blot		
2	To inspect the potential roles of circ_0004712 in E2-induced EMT in EM development	EM	1. Ishikawa cells	1. Cells were treated with 10^–8^ mol/L E2 for 48 h	1. Circ_0004712 knockdown repressed E2-induced cell migration activity in transwell assay	Upregulating circ_0004712 resulted in E2-induced cell migration *via* EMT event and the molecular mechanism might be correlated with β-catenin pathway in EM development	He et al. (2020)
			2. End1/E6E7 cells	2. Cells were transfected with siRNA of circ_0004712 and miR-148a-3p mimics	2. In dual-luciferase assay, direct binding sites between circ_0004712 and miR-148a-3p as well as miR-148a-3p and SOS2 were presented, suggesting miR-148a-3p targeted SOS2 and bound by circ_0004712 to regulate E2-induced EMT in endometrial epithelial cells		
					3. Transwell assay demonstrated that impeding circ_0004712 or enhancing miR-148a-3p expression significantly inhibiting E2-induced migration activity in cells		
3	To explore the molecular mechanism of LXA_4_ in inhibiting E2-induced EMT in EM	EM	Eutopic endometrial epithelial cells	Cells were treated with 200 nM E2 and 100 nM LXA_4_ for 48 h	1. Cells appeared spindle-shape and fibroblast-like after E2 treatment. Co-treatment of E2 and LXA_4_ reversed the morphological appearance change as observed in phase-contrast microscopy	LXA_4_ markedly repressed E2-enhanced EMT progress through ALXR-dependent manner, causing downregulation of ERK and p38 MAPK phosphorylation, thus suppressing EM development	Wu et al. (2018b)
					2. LXA_4_ abrogated the effects in mRNA and protein expressions of E2-induced of vimentin, N-cadherin and ZEB1 as well as E2-reduced E-cadherin as shown in qRT-PCR and Western blot		
					3. Cell migration and invasion assays revealed that LXA_4_ reduced E2-stimulated invasion and migration in cells		

E2, oestrogen; EMT, epithelial-mesenchymal transition; EM, endometriosis; NRP1, neurophilin 1, RT-PCR, real-time polymerase chain reaction; CDH1, cadherin-1; ACTA2, actin alpha 2; α-SMA, alpha-smooth muscle actin; SOS2, son of sevenless homolog 2; LXA_4_, lipoxin A_4_; ZEB1, zinc finger E-box-binding homeobox 1; qRT-PCR, quantitative real-time polymerase chain reaction; ALXR, lipoxin A_4_ receptor; p38 MAPK, p38 mitogen-activated protein kinase.

## 4 Discussion

**FIGURE 2:**
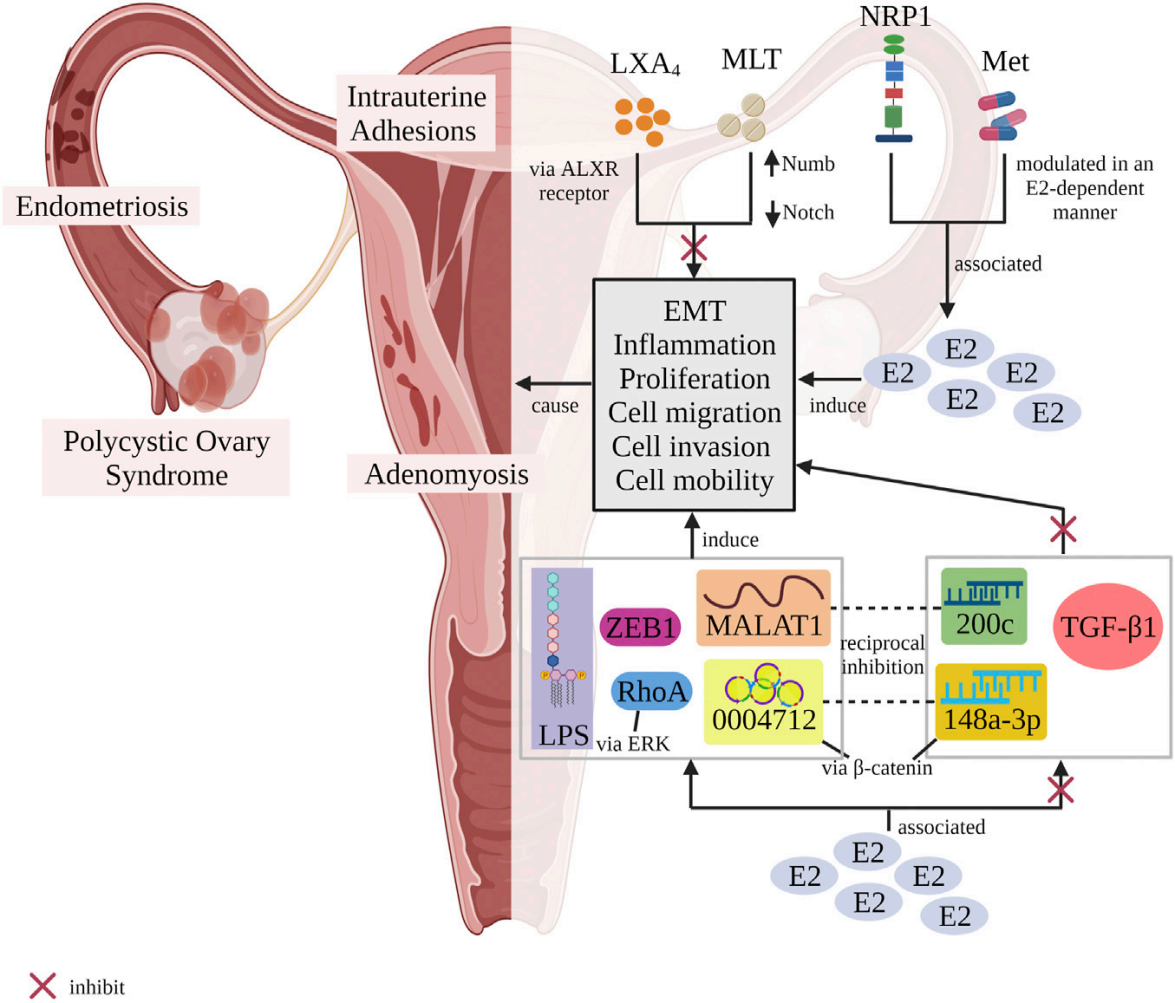
Schematic illustrating the potentiality of different stimuli in regulating EMT process with or without the association of E2 resulting in diseases-related female reproductive tract. LXA_4_ inhibited EMT *via* ALXR-dependent manner. Melatonin upregulated Numb expression and decreased the activity of Notch1 signalling pathway to block EMT occurrence. In contrast, both NRP1 and metformin were associated to E2 in regulating EMT. Metformin modulated EMT in an E2-dependent manner. Besides that, E2 was correlated to TGF-β1, miR200c and miR-148a-3p in suppressing EMT event while upregulated MALAT1, circ_0004712, ZEB1, LPS and RhoA (*via* activating ERK signalling pathway) to induce EMT process. There were two pairs of stimuli, MALAT1/miR200c as well as circ_0004712/miR-148a-3p which possessed an antagonism effect between them. An example, the antagonism effect happened when circ_0004712 sponged miR-148a-3p to activate β-catenin signalling pathway, thus promoting EMT event. E2, oestrogen; EMT, epithelial-mesenchymal transition; LXA_4_, lipoxin A_4_; MLT, melatonin; NRP1, neurophilin 1; Met, metformin; Numb and Notch, Notch1/Numb signalling pathway; ALXR, lipoxin A_4_ receptor; 200c, miR200c; 0004712, circ_0004712; 148a-3p, miR-148a-3p; LPS, lipopolysaccharide; β-catenin, β-catenin signalling pathway; ERK, ERK signalling pathway; TGF-β1, transforming growth factor-β1; ZEB1, zinc finger E-box-binding homeobox 1; RhoA, transforming protein RhoA; MALAT1, metastasis-associated lung adenocarcinoma transcript 1 (Designed with BioRender: https://biorender.com/).

The full search and complete assessment of numerous studies provided 10 articles correlated to E2 and EMT in the female reproductive system. A further assessment of these articles divulged the feasibility of varying stimuli in mediating EMT activities to initiate, support or hamper respective diseases. E2 is widely known for its contributions in the development and regulation of female reproductive functions (Findlay et al., 2010; Tang et al., 2019), yet little is known about its capacity to modulate EMT in the female reproductive tract. Together, this review assessed the roles of external stimuli related to E2 in mediating EMT in the development of diseases that ordinarily arise in the female reproductive system, typically EM (Figure 2).

### 4.1 Epithelial-to-mesenchymal transition in endometriosis

#### 4.1.1 EMT activation

EMT upregulation is one of the essential molecular mechanisms in disease development. It is defined as an increase in the expression of mesenchymal traits in cells undergoing plastic transformation from an epithelial state (Eades et al., 2011). This process is characterized by the disintegration of epithelial cell-cell junctions, loss of apical-basal polarity, rearrangement of cytoskeletal composition, changes in cell shape and morphology, increased cell protrusions and motility, loss of epithelial markers, and the activation of mesenchymal phenotype-inducing genes (Lamouille et al., 2014). In addition to signalling pathways and membrane receptors, the upregulation of EMT necessitates activation from external stimuli (Thiery et al., 2009). The Rho family, part of the Ras superfamily, encompasses small signalling proteins such as guanosine triphosphatases (Heo and Meyer, 2003). Huang *et al.* attempted to demonstrate the role and molecular mechanism of the Rho family on the E2 pathway, as well as proliferation and EMT in EM (Huang et al., 2020). They demonstrated the attenuation of E-cadherin and increased vimentin as well as N-cadherin protein expression in transforming protein RhoA (RhoA)-overexpressed eutopic endometrial epithelial cells. Moreover, other assays used in their study confirmed that the induction of RhoA resulted in increased cell migration, cell division and proliferation. In contrast, loss of RhoA function by utilising siRNA suppressed all these effects. In addition, RhoA has been shown to have an effect of E2 in terms of promoting EMT in EM. Likewise, Tantengco and others deduced that E2 apparently supports EMT in the migration and remodelling of cervical cells in the presence of an infection stimulus such as lipopolysaccharide (Tantengco et al., 2021). E2 was also shown to induce zinc finger E-box-binding homeobox 1 (ZEB1), a transcription factor related to EMT, in a study conducted by Wu *et al.* (Wu R.-F. et al., 2018). This evidence shows that EMT could be upregulated in EM, not only by the modulation of transcription factors, but also by the induction of external stimuli.

#### 4.1.2 Manipulation of EMT activation

Apart from the possible EMT inducers and EMT inhibitors, there are other stimuli that alter EMT in endometrial cells depending on the manipulated variables. An example of these manipulated variables is the co-treatment of cells with E2. Hu and others investigated treatment with metformin (a medication used to treat PCOS) alone or in combination with E2 in cultured human PCOS endometrial tissues and found inconsistently altered epithelial and mesenchymal markers (Hu M et al., 2020). For instance, there was an increase in the protein level of zinc finger protein SNAI1 (Snail) and decreased protein levels of claudin 1, N-cadherin and alpha-smooth muscle actin (α-SMA) when endometrial tissues were treated with metformin only. In combination with E2 treatment, the protein levels of cytokeratin 8 and Snail increased, whereas the protein levels of claudin 1, zonula occludens-1, zinc finger protein SNAI2 (Slug) and α-SMA decreased, suggesting that metformin might regulate endometrial EMT in an E2-dependent manner.

A similar study by Du et al. incorporated E2 treatment to examine endometrial EMT in both human endometrial epithelial cells and Ishikawa cells using metastasis associated lung adenocarcinoma transcript 1 (MALAT1), which is another class of long non-coding RNAs and miR200 family, which consists of non-coding microRNA members. Their study showed that the RNA expression levels of miR200s were downregulated while MALAT1 was upregulated in endometrial epithelial cells treated with E2 (Du et al., 2019). Also, they found that MALAT1/miR200s were associated to EMT, cell proliferation, growth, apoptosis, migration and invasion. This evidence not only demonstrates that E2 could alter MALAT1/miR200s expression to differentially regulate EMT in EM, but also implicates the suitability of MALAT1/miR200s as a diagnostic and prognostic biomarker of diseases. This is due to an antagonism effect between MALAT1 and miR200c found in Ishikawa cells when MALAT1 inhibition attenuated miR200c inhibitor-enhanced overexpression of EMT-associated markers (ZEB1, ZEB2 and vimentin) while miR200c inhibition increased the expression levels of siRNA-MALAT1-downregulated EMT-associated markers. The sponge effect of MALAT1 on miR200c has been a research subject in investigating the pathogenesis and progression of multiple diseases, including EM (Li et al., 2016; Liang et al., 2017; Pa et al., 2017), indicating a new therapeutic strategy.

#### 4.1.3 Targeting EMT inhibition in halting disease progression

It has been demonstrated that EMT instigates pathological processes in the normal epithelial cells of reproductive organs, leading to EM, adenomyosis and metastasis (Bilyk et al., 2017). Thus, extensive research has been performed to ameliorate related diseases by means of targeting EMT (Chen et al., 2020; Roy et al., 2021). However, EMT is connected to a vast number of processes and many signalling pathways may overlap (Cieślik et al., 2013). Therefore, targeting a specific orchestrator of EMT could improve the sensitivity in terms of halting disease progression. Transforming growth factor beta 1 (TGF-β1) is a secreted polypeptide cytokine. It is a prominent target in disease research as it controls myriad cellular responses during human development. Additionally, the TGF-β1 signalling pathway has been the most attentively defined pathway in inducing EMT (Gonzalez and Medici, 2014), as it activates the transcription of EMT-targeted genes, for example Snail, ZEB, forkhead box protein C2, high-mobility group AT-hook 2, paired related homeobox 1 and TWIST1 (Lamouille et al., 2014). Cao *et al.* found that treatment with TGF-β1 in an IUA cell model decreased the expression of epithelial markers and increased the expression of mesenchymal markers; interestingly, the effects were reversed when cells were pre-treated with E2 (Cao et al., 2020).

Previously, melatonin, a hormone that controls sleep-wake cycle, was shown to inhibit TGF-β1-induced EMT in A549 cells, which are adenocarcinomic human alveolar epithelial cells (Yu et al., 2016). This study found that melatonin is associated with TGF-β1 in inhibiting EMT. Moreover, melatonin abolished E2-induced cell migration, cell invasion and EMT by modulating Notch1/Numb expression in EM epithelial cells (Qi et al., 2018). This study futher verified that melatonin might be an ideal therapeutic in attenuating EMT to restrain the development of EM. Olive extracts from *Olea europaea* have also been shown to prevent EMT in human nasal respiratory epithelial cells by retaining a cuboidal cell shape, with higher expression of E-cadherin and lower expression of vimentin (Razali et al., 2018). Ginsenoside Rh2, a bioactive component in ginseng, has shown unexpected abilities in apoptosis induction and EMT inhibition in two types of human endometrial cell lines, HEC1A and Ishikawa cells (Kim et al., 2017). The results show that there were significant increases in the number of apoptotic cells and the E-cadherin/tubulin ratio as well as notable decreases in the vimentin/tubulin ratio, cell invasion rate and cell migration ability. These studies attest that even natural bioactive compounds can inhibit EMT in diseased cells.

### 4.2 Modulation and regulation of E2-induced EMT

It is compelling that E2 promotes EMT in a number of E2-dependent diseases like EM and adenomyosis. Hence, researchers tend to induce EMT activities by treating targeted cells with E2 prior to further analysis of a disease. An essential mechanistic link between E2 and EMT is circular RNA (circRNA), which is a long non-coding RNA (lncRNA) capable of regulating genes at the transcriptional or post-transcriptional level (Qu et al., 2015); these molecules act as an miRNA ‘sponge’ to inhibit the activity of one or multiple miRNAs (Yu and Kuo, 2019). Evidence of circRNA regulated E2-induced EMT was presented in a study by He *et al.* using Transwell assays, where knockdown of cir_0004712 coupled with overexpression of miR-48a-3p significantly repressed cell migration in E2-treated endometrial epithelial cells; this effect was recovered with the inhibition of miR-48a-3p (He et al., 2020). This result shows that circ_0004712 could bind to miR-148a-3p to mediate E2-induced EMT in EM.

Another stimulus that could modulate E2-induced EMT is the protein receptor neurophilin 1 (NRP1). Hu and others showed that silencing NRP1 clearly restored the expression of E-cadherin, abolished the expression of α-SMA and impaired cell migration in E2-induced endometrial cells (Hu R et al., 2020). This result indicates that NRP1 might be a potential therapeutic for adenomyosis patients as it could modulate E2-induced EMT in endometrial cells. Lipoxin A_4_ (LXA_4_), a pro-resolving and anti-inflammatory molecule, was investigated for its effects on EMT in E2-induced eutopic endometrial epithelial cells. Based on a study by Wu *et al.*, combined treatment with E2 and LXA_4_ reversed the morphological change in cells that initially appeared spindle-shape and fibroblast-like when treated with E2 alone (Wu R. F. et al., 2018). Also, LXA_4_ has been shown to reduce the effect of E2-induced migration and invasion in these cells. Since LXA_4_ competes with E2 to bind to ER (Russell et al., 2011), these results suggest that LXA_4_ could repress EM development by occupying ER to inhibit E2 signalling, thereby averting EMT in endometrial cells.

### 4.3 Crosstalk between Wnt, E2 and EMT: Is this related to MRKH syndrome ?

Vaginal agenesis is a congenital disorder whereby the vagina does not develop. This rare disorder is commonly seen in young girls with cervical agenesis and MRKH syndrome (Nakhal and Creighton, 2012). In some cases, the signs and symptoms are identified when patients are evaluated for primary amenorrhea with otherwise typical growth and pubertal development (Fontana et al., 2017). To treat MRKH syndrome, a two-step procedure is used: creation of a vagina (neovagina) and subsequent anastomosis of the uterus with the newly created vagina. The use of *in vitro* cultured vaginal mucosa has emerged as a novel technique to epithelise the new vaginal wall in recent years. In this review, we assessed the involvement of Wnt4 and E2 on EMT in the female reproductive tract, specifically EM. Since information on these factors on vaginal agenesis is still lacking, we postulate that EMT, with the involvement of Wnt4 and E2, also occurs in the vagina as the endometrium extends downwards to vaginal tract.

Wnt4 has been shown to be crucial for female sexual development. Wnt4 protein suppresses male sexual differentiation by repressing the biosynthesis of gonadal androgen. Inhibition of Wnt4 has been studied by knocking out the Wnt4 gene in mice, resulting in failed Müllerian duct formation (Vainio et al., 1999). Wnt4 has also been studied with regard to its function in the development of the Müllerian duct, as mutations in or the absence of Wnt4 in the Müllerian mesenchyme results in vaginal agenesis (Biason-Lauber et al., 2004; Biason-Lauber et al., 2007; Prunskaite-Hyyryläinen et al., 2015; St-Jean et al., 2019), a deformity that is usually diagnosed in MRKH patients. One case study discussed a woman without structures derived from the Müllerian ducts (uterus and fallopian tubes), with unilateral renal agenesis and clinical signs of androgen excess (Biason-Lauber et al., 2007). In contrast, earlier studies reported that they detected no Wnt4 abnormalities in the DNA extracted from women with MRKH syndrome (Drummond et al., 2008; Ravel et al., 2009; Philibert et al., 2011), considering that the function of Wnt4 gene and the genotypic variability of MRKH are not well comprehended yet.

Numerous signalling pathways can initiate EMT in response to the presence of stimuli such as E2. One of the leading pathways associated with EMT is the Wnt signalling pathway (Zmarzły et al., 2020). Existing research on the effects of Wnt signalling in E2-induced EMT is scarce. From the assessment of the studies included in this review, very few studies were found to evaluate the relationships between Wnt proteins and EMT-induced cells, specifically in relation to diseases associated with the female reproductive tract. Despite that, there was one study conducted by Guo et al. (2014), who successfully showed the overexpression of Wnt4 gene upregulated the markers of EMT by the decreased expression of E-cadherin and increased expression of α-SMA. Previous study has demonstrated that the expression of Wnt4 is directly regulated by E2, in an ERα-dependent manner in growth-hormone (GH)-producing cells (Miyakoshi et al., 2009). Another study showed that the number of Wnt4 mRNA copies increased with the injection of E2 in mice’s uteri. Also, a proposed model in the study suggested that E2 could lead to the activation of Wnt signalling by induction of Wnt4 expression in uterine epithelial cells. (Hou et al., 2004). These studies indicated that E2 and Wnt4 associate to each other and their interactions could result in the activation of EMT.

**FIGURE 3:**
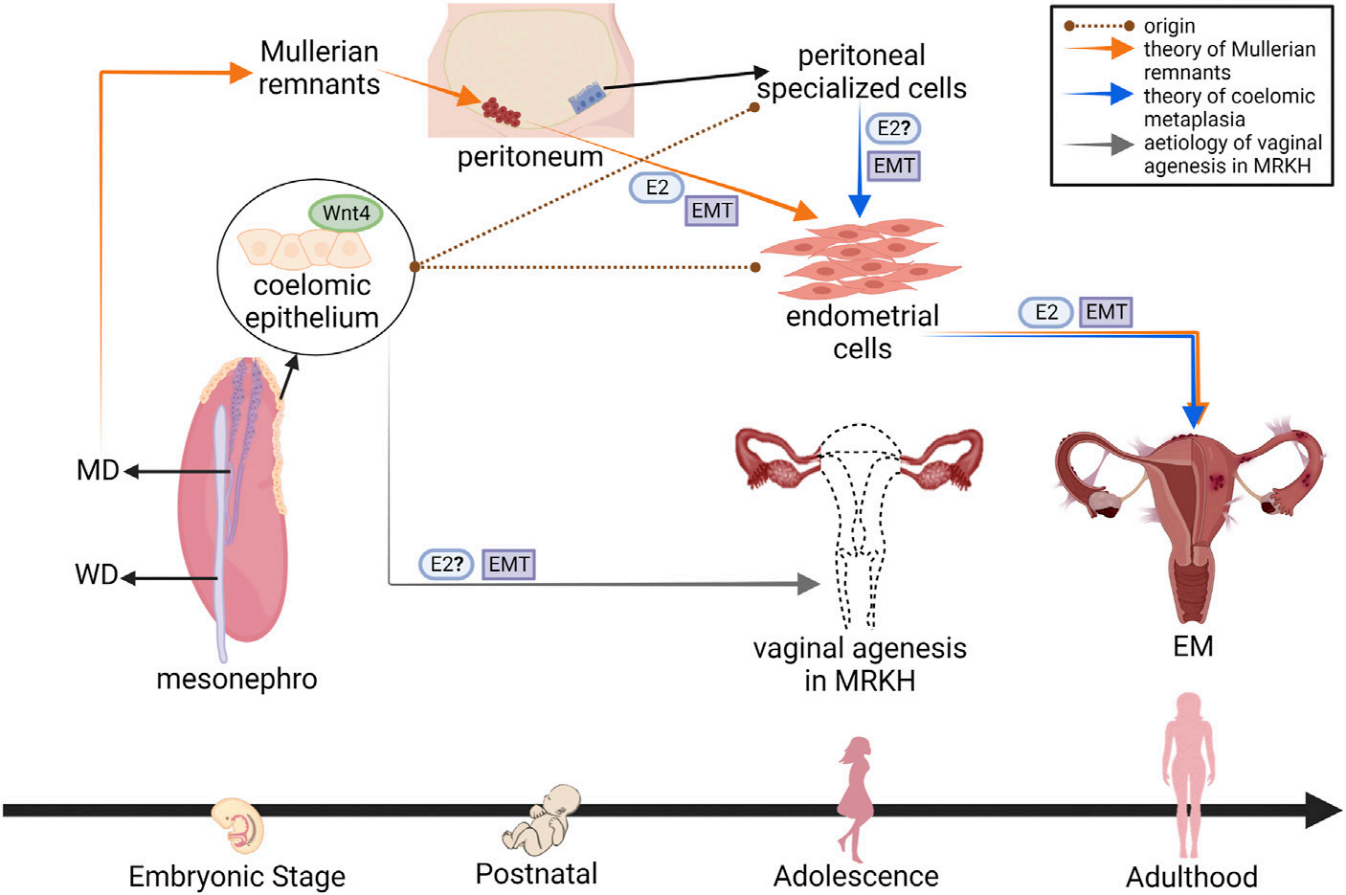
Crosstalk between E2, Wnt4 and EMT in the development of EM: Aetiology of vaginal agenesis in MRKH. Pathogenesis of EM has been classically defined to originate from Mullerian-related cells expressing Wnt4 in early human development. Under the influence of E2 and other appropriate stimuli, the cells exhibit EMT and progress into endometriotic lesions in adults. Corresponding to MRKH patients which also initiated from Mullerian cells, the disorder develops when Wnt4 is mutated in coelomic epithelium of embryo. This could then trigger EMT which leads to female reproductive organ formation failure, such as vagina and uterus. E2, oestrogen; EMT, epithelial-mesenchymal transition; Wnt4, Wnt family member 4; MD, Mullerian duct; WD, Wolffian duct; MRKH, Mayer-Rokitansky-Küster-Hauser syndrome; EM, endometriosis (Designed with BioRender: https://biorender.com/).

As reported, the role of EMT in EM development is possible *via* E2 induction but the association with vaginal agenesis in MRKH patients remains unrevealed. Figure 3 showed possible relations between EM and vaginal agenesis as those affected organs are descended from the primitive Mullerian duct and speculated to involve Wnt4-expressing Mullerian cells from the Mullerian development process. Many theories have been came up in explaining the pathogenesis of EM but remain to be proven. In particular, coelomic metaplasia: Specialized cells of peritoneum share common embryological lineage (from coelomic epithelium) as endometrial cells, develop into endometriotic lesions by metaplastic transformation (Gruenwald, 1942; Dhesi and Morelli, 2015). Mullerian remnants: These cells misplaced the primitive endometrial cells during organogenesis and implanted in their migratory path across the pelvic floor due to aberrant differentiation and migration, later induced into endometrial cells by specific stimuli (e.g., E2) (Mai et al., 1998). Those theories revealed that EMT involves in endometrial-like tissue formation as the Mullerian cells were found proliferated, migrated subsequently progressed into endometriotic lesions.

EMT induction factors that are closely related to EM have been discussed such as E2; involves in hormonal regulation, and Wnt4; Mullerian-expressing genes. Bilyk et al. (2017) and Baranov et al. (2018) stated that EM in adults might be associated to the early life of Mullerian-expressing genes in uterine endometrium. On the other hand, vaginal agenesis is developed when the Mullerian duct fails to form as a result of Wnt4 anomalies and other factors, potentially E2 and EMT as adopted from the theories of EM development. The Mullerian progenitors, coelomic epithelial cells, express Wnt4 for regulating Mullerian invagination and elongation. These Mullerian stages require Wnt4-expressing progenitor cells to undergo EMT and differentiate into Mullerian mesenchymal cells, enabling cranial-caudal cell migration of Mullerian duct in close proximity to Wolffian duct. Once Mullerian ducts fuse with urogenital sinus, the cells undergo MET and specialize into epithelial subtypes, giving rise to female reproductive tract constituting fallopian tubes, uterus, cervix and upper vagina (Bilyk et al., 2017; Santana Gonzalez et al., 2021). This supports earlier studies reporting vaginal agenesis occurs when there is deregulation of Wnt4 (Wnt4 mutation or deficiency) as well as the speculation of EMT (cells remain mesenchymal and not forming epithelial) during the development of Mullerian duct, though the role of E2 in this event is still elusive.

Thus, it could be assumed that E2 and Wnt4 associate in an undefined manner to induce EMT in the development of diseases in female reproductive tract such as EM as well as vaginal aplasia in MRKH patients. Still, further research has to be done to strongly validate how E2 regulate Wnt4 or Wnt signalling pathway to potentiate EMT in disease development. Collectively, this review will provide new insights into the mechanisms related to E2 and Wnt4 in regulating vaginal epithelialisation to ensure better surgical outcomes. We anticipate that this will become a key aspect in the surgical procedure for creating a new vagina (neovagina) in the near future.

## 5 Conclusion

In relation to the overall results discussed in this review, there is a constraint whereby the paucity of EMT in the non-cancer field limits complete understanding on the regulation of EMT processes in non-cancer related diseases. Hence, this review may provide a new perspective for non-cancer research, especially regarding reproductive-related studies. This review presents the potentials to influence EMT through various stimuli in diseases associated with the female reproductive system, chiefly EM. Additionally, E2 could also be an EMT regulator and is prone to be mediated by other EMT orchestrators to cause EMT, principally in endometrial epithelial cells. This review also highlighted the possible participation of Wnt and E2 in EMT in vaginal epithelial cells, which may result in the complete absence of vaginal structure, an abnormality observed in women with MRKH syndrome. Collectively, this overview could serve as a foundation to pursue ideal or novel targets for the treatment of diseases associated with the female reproductive system, namely vaginal agenesis, by regulating vaginal epithelialisation to ensure better surgical outcomes.

## Abbreviations

E2, Oestrogen; EM, Endometriosis; EMT, Epithelial-mesenchymal transition; ERα, Oestrogen receptor α; ERβ, Oestrogen receptor β; IUA, Intrauterine adhesion; LXA_4_, Lipoxin A_4_; MALAT1, Metastasis associated lung adenocarcinoma transcript 1; MRKH, Mayer-Rokitansky-Küster-Hauser syndrome; NRP1, Neurophilin 1; PCOS, Polycystic ovary syndrome; RhoA, Transforming protein RhoA; Snail, Zinc finger protein SNAI1; TGF-β1, Transforming growth factor beta 1; TWIST1, Twist-related protein 1; Wnt4, Wnt family member 4; ZEB, Zinc finger E-box-binding homeobox; α-SMA, Alpha-smooth muscle actin.
