# Supplementary material for: Oestrogen-induced epithelial-mesenchymal transition (EMT) in endometriosis: Aetiology of vaginal agenesis in Mayer-Rokitansky-Küster-Hauser (MRKH) syndrome
Source: Front Physiol. 2022 Dec 13;13:937988. doi: 10.3389/fphys.2022.937988 (PMC9793092; doi:10.3389/fphys.2022.937988)
Supplement: Supplementary file 1 [file Table1.docx]

Supplementary Material

**Supplementary Table 1.** Risk of bias assessment for included studies.

| References | | (Huang et al., 2020) | (Tantengco et al., 2021) | (Wu et al., 2018a) | (Cao et al., 2020) | (Qi et al., 2018) | (Hu et al., 2020a) | (Du et al., 2019) | (Hu et al., 2020b) | (He et al., 2020) | (Wu et al., 2018b) |
| --- | --- | --- | --- | --- | --- | --- | --- | --- | --- | --- | --- |
| Selection | Number across groups were matched | + | + | + | + | + | + | + | + | + | + |
|  | Concealment of exposure allocation | NA | NA | NA | NA | NA | NA | NA | NA | NA | NA |
|  | Appropriate comparison group | + | + | + | + | + | + | + | + | + | + |
|  | Absence of confounding factors | + | + | + | + | + | + | + | + | + | + |
| Performance | Identical experimental condition across groups | + | + | + | + | + | + | + | + | + | + |
|  | Blinded outcome assessor | NA | NA | NA | NA | NA | NA | NA | NA | NA | NA |
| Attrition | Outcome data were complete with no exclusion from analysis | + | + | + | + | + | + | + | + | + | + |
| Detection | Appropriate exposure assessment | + | + | + | + | + | + | + | + | + | + |
|  | Appropriate outcome assessment | + | + | + | + | + | + | + | + | + | + |
| Reporting | All measured outcomes were reported | + | + | + | + | + | + | + | + | + | + |
